# Molecular Survey of *Trypanosoma*, *Babesia* and *Theileria* Infections in Major Livestock and Rodents in Xinjiang, Northwestern China

**DOI:** 10.3390/vetsci13080833

**Published:** 2026-08-19

**Authors:** Yongchang Li, Xianyue Fu, Zihan Wang, Iqra Zafar, Zeyun Cui, Shiyun Li, Xiujuan Feng, Sen Yang, Ercha Hu, Chahan Bayin

**Affiliations:** 1 Parasitology Laboratory, Veterinary College, Xinjiang Agricultural University, Urumqi 830052, China; 2 Laboratory of Sustainable Animal Environment, Graduate School of Agriculture Science, Tohoku University, Sendai City 9820023, Japan

**Keywords:** Xinjiang, cattle, camels, horses, mouse, *Trypanosoma*, *Babesia*, *Theileria*, epidemiology

## Abstract

From September 2024 to September 2025, 310 blood samples from cattle (*n* = 151), camels (*n* = 44), horses (*n* = 71), and wild mice (*n* = 44) across six Xinjiang counties/cities were screened by microscopy, genus-specific PCR, sequencing, and phylogenetic analysis. Overall, 46.77% (145/310) of the samples were positive for haemoprotozoan infection. *Trypanosoma* spp. showed the highest prevalence (25.48%, 79/310), followed by Theileria (16.13%, 50/310) and Babesia (5.16%, 16/310). Notable host-specific patterns emerged: camels had the highest *T. evansi* occurrence (86.36%), while wild mice had the highest Theileria prevalence (75%). Dual infections (mostly Trypanosoma + Theileria) occurred in 8.39% (26/310) of animals, and a triple infection was found in 0.32% (1/310). Phylogenetic analysis identified *T. evansi* as predominant trypanosome, clustering with isolates from India and Saudi Arabia; the major piroplasms identified were *T. annulata*, *T. equi*, and an unidentified *Babesia* sp. This study represents the first multi-host molecular survey in Xinjiang, revealing a high infection burden, diverse parasite species, and frequent co-infections.

## Simple Summary

This survey investigated haemoprotozoan infections in cattle, camels, horses, and wild mice across Xinjiang, China. Among 310 animals examined, nearly half (46.8%) were infected with at least one haemoprotozoan parasite. Trypanosoma was the most prevalent genus (25.5%), followed by Theileria (16.1%) and Babesia (5.2%). Infection rates varied widely among hosts: camels showed the highest prevalence of Trypanosoma infection (86.4%), while wild mice were the most frequent carriers of *Theileria* (75%). Co-infections were also commonly observed. The identified species included *Trypanosoma evansi* (*T*. *evansi*), *Theileria annulata* (*T. annulata*), and *Theileria equi* (*T. equi*). Being a multi-host molecular survey conducted in Xinjiang, this study reveals a substantial pathogen burden and provides baseline data for future surveillance and tailored control measures.

## 1. Introduction

Xinjiang Uygur Autonomous Region, situated along China’s northwestern frontier, is one of the country’s major pastoral livestock production regions. Its extensive grasslands hold strategic importance for both national ecological security and the regional livestock economy [1]. Cattle, camels, and horses represent the backbone of local herbivorous livestock farming, serving as indispensable productive assets and primary sources of income for local farmers and herders. Livestock production relies largely on seasonal rotational grazing and free-range management, which results in prolonged animal exposure to natural environments and arthropod vectors [2]. Additionally, global climate change has altered pastoral ecosystems, contributing to shifts in the distribution and epidemiology of livestock diseases [3].

Among the major diseases affecting livestock, haemoprotozoan infections caused by *Trypanosoma* spp., *Babesia* spp., and *Theileria* spp. pose a significant threat to animal health. These parasites infect a wide range of hosts through diverse arthropod vectors. Because infections are frequently subclinical or present with non-specific signs, their detection and control remain particularly challenging [4]. Consequently, they impair animal health and productivity, thereby undermining the long-term sustainability of herbivorous livestock production systems [5,6]. Trypanosomes are mechanically transmitted by haematophagous insects such as tabanids and stable flies. Trypanosomiasis is characterized by high fever, anaemia, weight loss, and occasional mortality. Among the *Trypanosoma* species, *T. evansi* exhibits the broadest host range, infecting camels, cattle, horses, and numerous other domestic and wild animals [7,8]. In contrast, Babesia and Theileria species are tick-borne piroplasms transmitted by ixodid vectors. They target different host cells: Babesia primarily invades erythrocytes (red blood cells), while Theileria mainly targets lymphocytes (white blood cells). Infections lead to haemolytic anaemia, jaundice, and lymphadenopathy, with severe cases often proving fatal [9,10]. These three pathogen groups are widely distributed across the globe and remain endemic in many tropical and subtropical regions, particularly in Africa, Asia, and the Middle East [11,12].

Between 2020 and 2025, most haemoprotozoan epidemiological studies in China focused on a single pathogen or host species. Consequently, integrated investigations involving multiple host species and haemoprotozoan parasites remained limited. In the current livestock production systems of Xinjiang, cattle, camels, horses, and other domestic animals are frequently managed together in mixed herds and co-graze on shared pastures. This husbandry practice may facilitate pathogen transmission among different host species through shared arthropod vectors. The widespread presence of wild rodents further contributes to pathogen maintenance and the occurrence of mixed infections [13,14]. Mixed infections can exacerbate disease severity, increase the risk of mortality, complicate diagnosis, and reduce the effectiveness of disease control programmes [15]. Moreover, Xinjiang has a heterogeneous climate, featuring deserts, oases, and grasslands, that provide suitable habitats for insect vectors and ixodid ticks [16]. Nevertheless, comprehensive epidemiological studies that simultaneously detect Trypanosoma, Babesia, and Theileria in cattle, camels, horses, and wild rodents in Xinjiang are still lacking. This leaves a critical gap in our understanding of the epidemiology and transmission of these parasites in this major pastoral region.

To address these overlapping transmission risks, we evaluated blood samples collected from cattle, camels, horses, and wild mice across six representative pastoral areas in Xinjiang. We employed a diagnostic workflow combining Giemsa-stained blood smear microscopy, genus- and species-specific PCR assays, sequencing, and phylogenetic analysis to simultaneously detect the three major hemoprotozoan pathogens. Specifically, this study aimed to characterize the overall infection rate, host distribution patterns, co-infection profiles, and the phylogenetic relationships among the dominant species. The findings are expected to provide a scientific foundation for evidence-based surveillance, the development of prevention and control measures, and improved animal disease management in the region.

## 2. Materials and Methods

### 2.1. Reagents and Equipment

The 2× Taq Master Mix, Whole Blood Genomic DNA Extraction Kit, Agarose Gel DNA Recovery Kit (Enhanced), and 2K DNA Marker were purchased from Tiangen Biotech Co., Ltd. (Beijing, China). The electrophoresis apparatus (DYY-6C) was obtained from Beijing Liuyi Biotechnology Co., Ltd. (Beijing, China), and gel images were captured using a gel imaging system (Bio-Rad Laboratories, Inc.,Hercules, CA, USA). Giemsa staining reagent was purchased from Beijing Solarbio Science & Technology Co., Ltd. (Beijing, China). Blood smears were examined using a TE2000 optical microscope (Nikon Corporation, Tokyo, Japan).

### 2.2. Sample Collection

**Figure 1:**
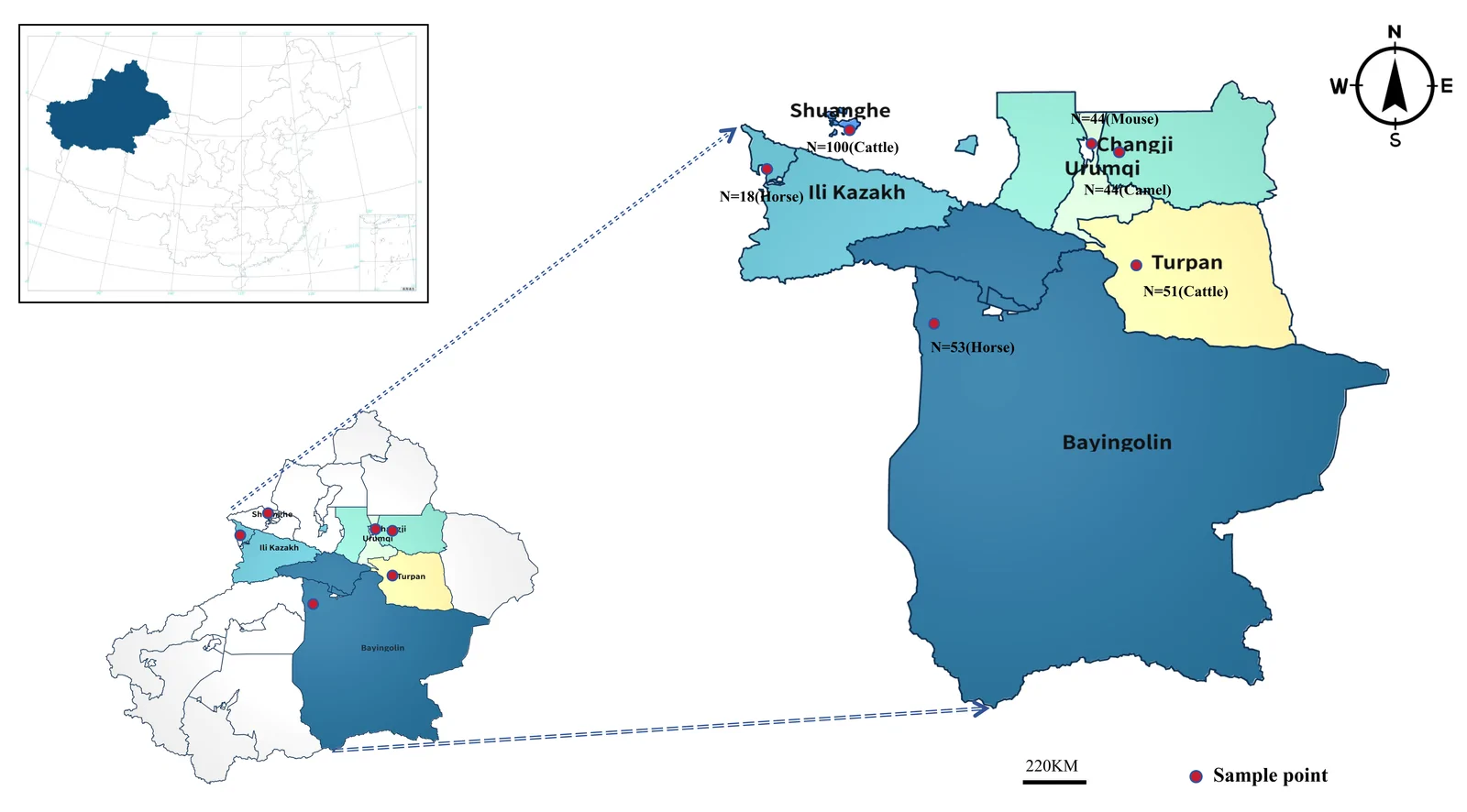
Geographic distribution of sampling sites and sample sizes across Xinjiang, China. Sampling locations for cattle, camels, horses, and wild mice included in this study. The inset map (Upper left) shows the location of Xinjiang within China. The enlarged map illustrates the six sampling regions in Xinjiang: Urumqi (Camel, *n* = 44), Changji (Mouse, *n* = 44), Turpan (Cattle, *n* = 51), Bayingolin (Horses, *n* = 53), Shuanghe (Cattle, *n* = 100), and Yili Kazakh Autonomous Prefecture (Horses, *n* = 18). Red dots indicate sampling sites. Total sample sizes for each host species are shown on the map.

From September 2024 to September 2025, a total of 310 blood samples were collected from diverse pastoral locations in Xinjiang, China, including Midong District, Turpan City, Shuanghe City, Ili, and Changji City (Figure 1). The study included cattle, camels, horses, and wild mice that were either clinically healthy or suspected of hemoprotozoan infection. From each animal, 1.5–5 mL of whole blood was collected into tubes containing EDTA and then stored at 4 °C. Both blood smear preparation and genomic DNA extraction were completed within 3 days of sample collection. This study was conducted in accordance with the ethical guidelines of Xinjiang Agricultural University. All animal procedures were approved by the Institutional Animal Care and Use Committee (IACUC) of Xinjiang Agricultural University (Approval No. 2023052).

### 2.3. Giemsa-Staining and Microscopic Examination

Thin blood smears were prepared from fresh anticoagulated blood, air-dried, fixed with methanol, stained with Giemsa, rinsed with running water, air-dried, and examined under a light microscope [17]. The smears were specifically examined for the presence of *Trypanosoma*, *Babesia*, and *Theileria* parasites in erythrocytes and plasma, and microscopic findings were recorded.

### 2.4. DNA Extraction

Genomic DNA was extracted from all blood samples (*n* = 310) using a whole-blood genomic DNA extraction kit, according to the manufacturer’s instructions. The extracted DNA was quantified and stored at −20 °C for further analysis.

### 2.5. PCR Amplification

Extracted DNA was subjected to PCR amplification to detect *Trypanosoma* spp., *Babesia* spp., and *Theileria* spp. using the primers listed in Table 1. Each PCR reaction was performed in a total volume of 13 μL, consisting of 0.5 μL each of forward (10 uM) and reverse primers, 6.5 μL of 2× PCR master mix, 4.5 μL of ddH_2_O, and 1 μL of DNA template (25–30 ng/μL). The amplified products were analysed by electrophoresis on a 1.0% agarose gel [18], and the results were visualised using a gel imaging system.

### 2.6. Sequence Determination and Phylogenetic Analysis

Amplicons of the expected target sizes were purified using an Enhanced Agarose Gel DNA Recovery Kit (Tiangen Biotech, Beijing, China). The purified products were sent to Sangon Biotech (Shanghai, China) for Sanger sequencing. The resulting nucleotide sequences were compared against the GenBank database using NCBI BLAST (https://blast.ncbi.nlm.nih.gov/Blast.cgi). Phylogenetic trees were constructed using the Neighbor-Joining method in MEGA (v. 11) software, with bootstrap support evaluated using 1000 replicates [24].

## 3. Results

### 3.1. Microscopic Examination of Blood Smears

**Figure 2:**
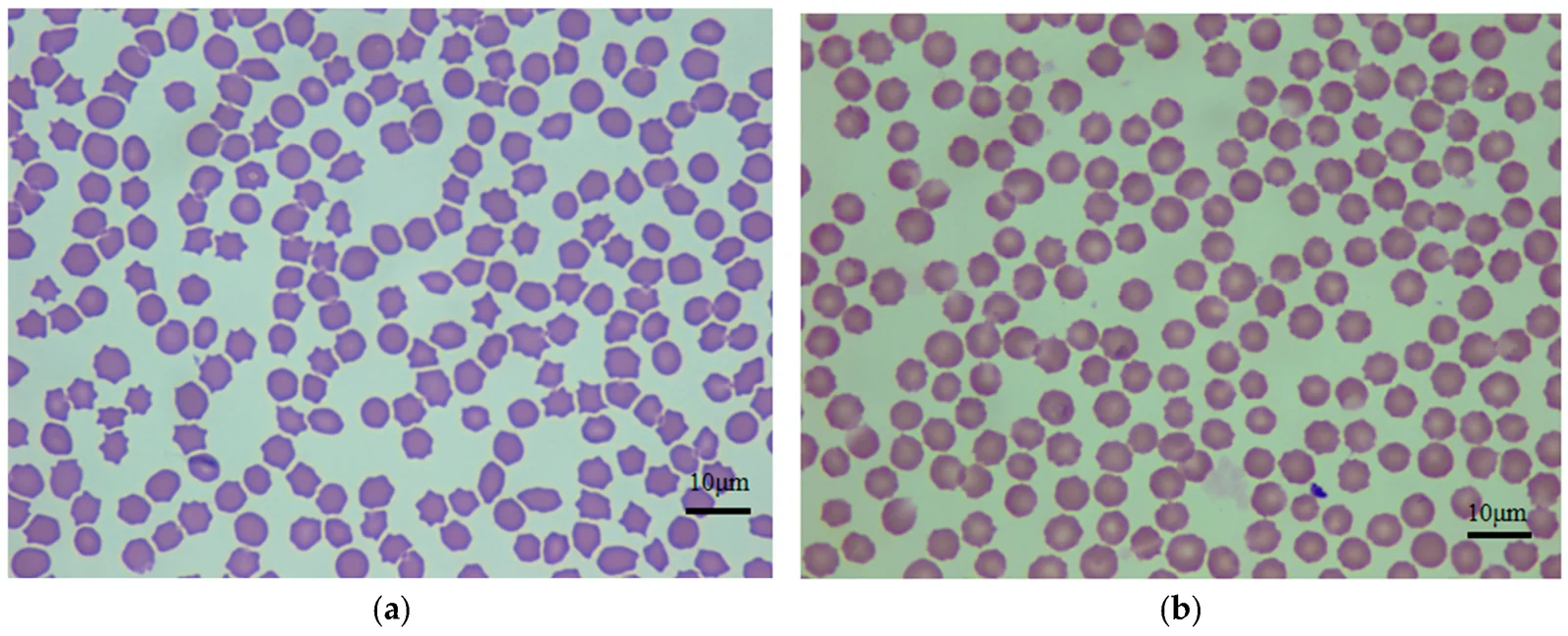

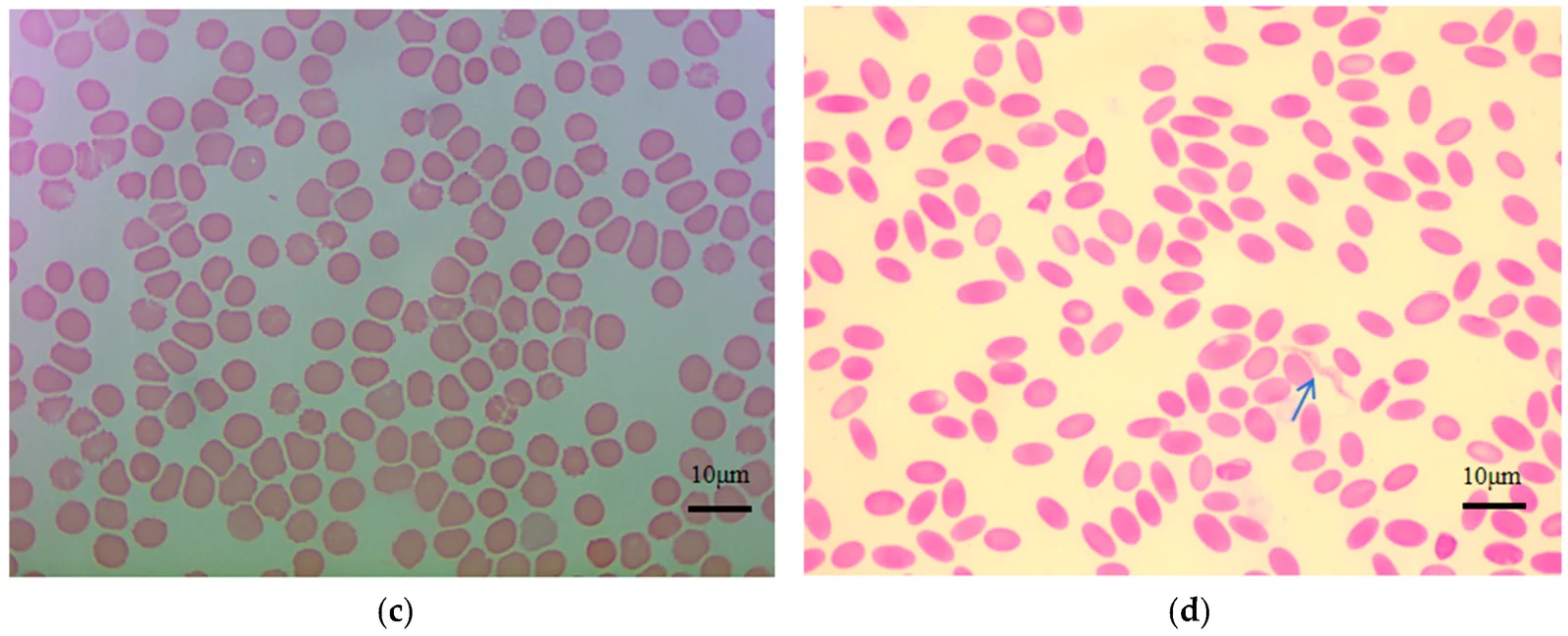
Representative Giemsa-stained thin blood smears across different host species under light microscopy (10 × 100 nm oil immersion objective) (**a**). Horse blood smear showing no detectable haemoprotozoan parasites, displaying normal erythrocyte morphology; (**b**). Cattle blood smear showing no detectable haemoprotozoan parasites; (**c**) Negative blood smear from a wild murine host (mice) demonstrating clean intercellular spaces; (**d**). Positive blood smear from a camel host showing extracellular *Trypanosoma* spp. parasites with characteristic slender, elongated morphology located between red blood cells. The Trypanosome is indicated by the blue arrow.

Microscopic examination of Giemsa-stained blood smears did not reveal parasites in cattle (Figure 2a), horses (Figure 2b), or wild mice (Figure 2c). However, trypanosome-like parasites were detected in four camel blood samples (Figure 2d). The protozoa were observed in the plasma between erythrocytes. Their morphological features were consistent with members of the genus Trypanosoma, appearing as slender, elongated, willow-leaf or fusiform bodies with tapered ends and a distinct free flagellum.

### 3.2. PCR Test and Gel Imaging Results

#### 3.2.1. Baseline Pathogen Prevalence and Host Distribution

**Figure 3:**
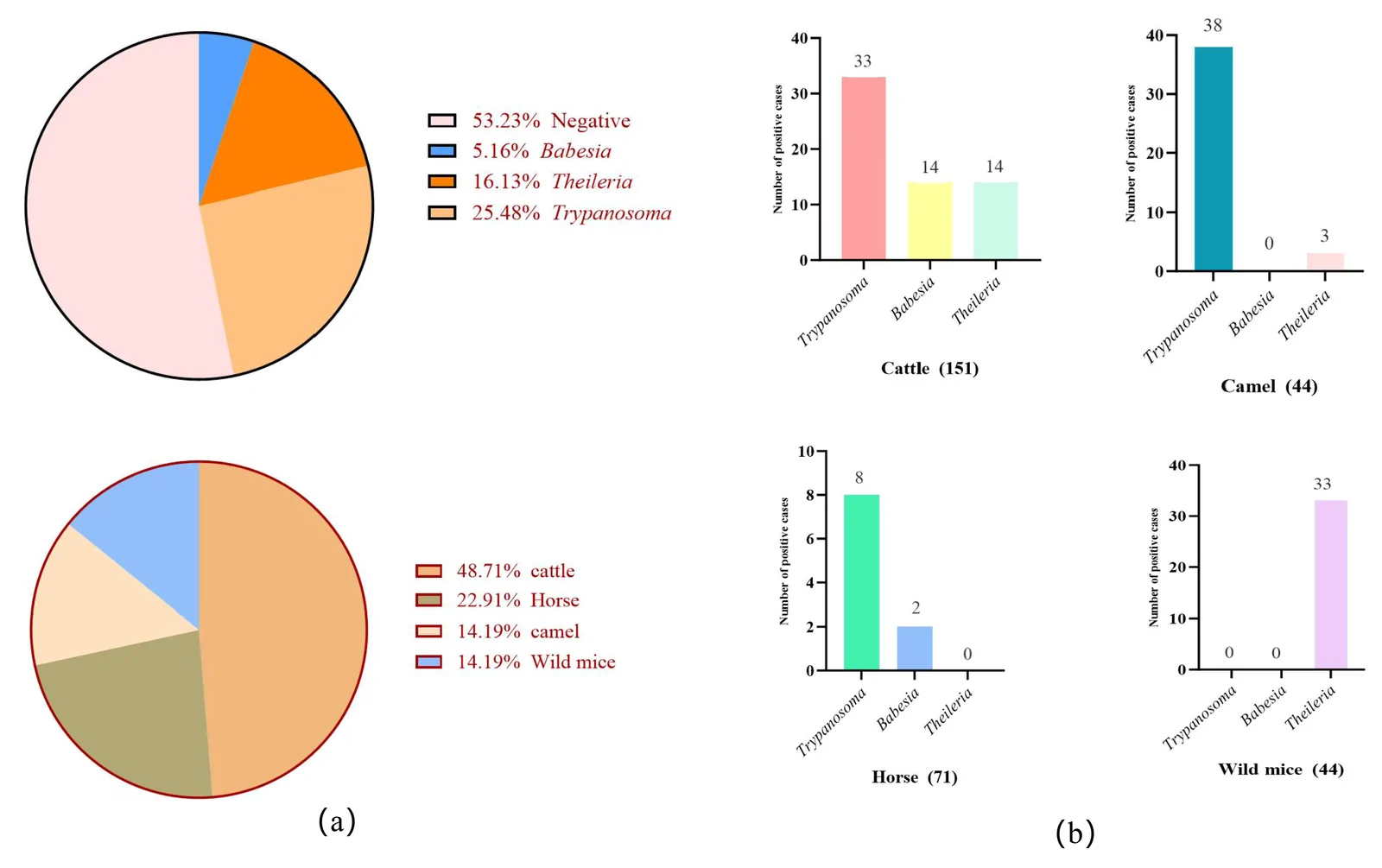
Overall molecular prevalence, sample composition, and host–pathogen distribution profiles. (**a**): Overall PCR positivity rates and host composition: the top pie chart illustrates the total baseline prevalence of Trypanosoma (25.48%), Theileria (16.13%), and Babesia (5.16%) across all examined animals alongside negative cases (53.23%); the bottom pie chart displays the relative proportions of the sampled host species (48.71% cattle, 22.91% horse, 14.19% camel, and 14.19% wild mice). (**b**): Number of Trypanosoma, Babesia, and Theileria infections detected in cattle (*n* = 151), camels (*n* = 44), horses (*n* = 71), and wild mice (*n* = 44).

Among the 310 blood samples tested, the overall PCR-based prevalence of blood protozoan parasites was 46.77% (Figure 3a), and the test results for the three pathogens are different in different animals (Figure 3b). The specific molecular prevalence rates for *Trypanosoma* spp., *Babesia* spp., and *Theileria* spp. were 25.48%, 5.16%, and 16.13%, respectively. When analyzed by host species, overall infection rates were 40.40% in cattle, 14.08% in horses, 95.35% in camels, and 75% in wild mice.

#### 3.2.2. Regional Variation in Infection Rates

Molecular screening across the six sampled regions in Xinjiang revealed significant geographic variations in pathogen distribution. Urumqi (including Midong District) exhibited the highest Trypanosoma positivity rate at 86.36% (38/44), whereas no *Trypanosoma* DNA was detected in Changji or Bayingolin. Turpan showed positivity rates of 25.5% for both Babesia and Theileria. In contrast, Theileria infections were absent in samples collected from Ili or Bayingolin (Table 2).

#### 3.2.3. Single and Mixed Infections Across Host Species

Among the Trypanosoma-positive samples (79/310, 25.48%), camels had the highest prevalence (86.36%, 38/44), while no single infections were detected in wild mice. For Theileria (50/310, 16.13%), wild mice showed the highest prevalence (75%, 33/44), whereas horses had no Theileria infections. Babesia was detected in 16 samples (5.16%), with the highest prevalence in cattle (14/151, 9.27%), whereas no infections were detected in wild mice and camels. A total of 26 dual infections (8.39%) were identified, comprising Trypanosoma + Theileria (4.52%, 14/310) or Trypanosoma + Babesia (3.87%, 12/310) combinations, with cattle representing the most frequently co-infected host species. A single case of triple infection (Trypanosoma + Theileria + Babesia) was detected in a bovine sample (0.32%); no triple infections were observed in any other host species (Table 3).

### 3.3. Sequencing and Phylogenetic Analysis

**Figure 4:**
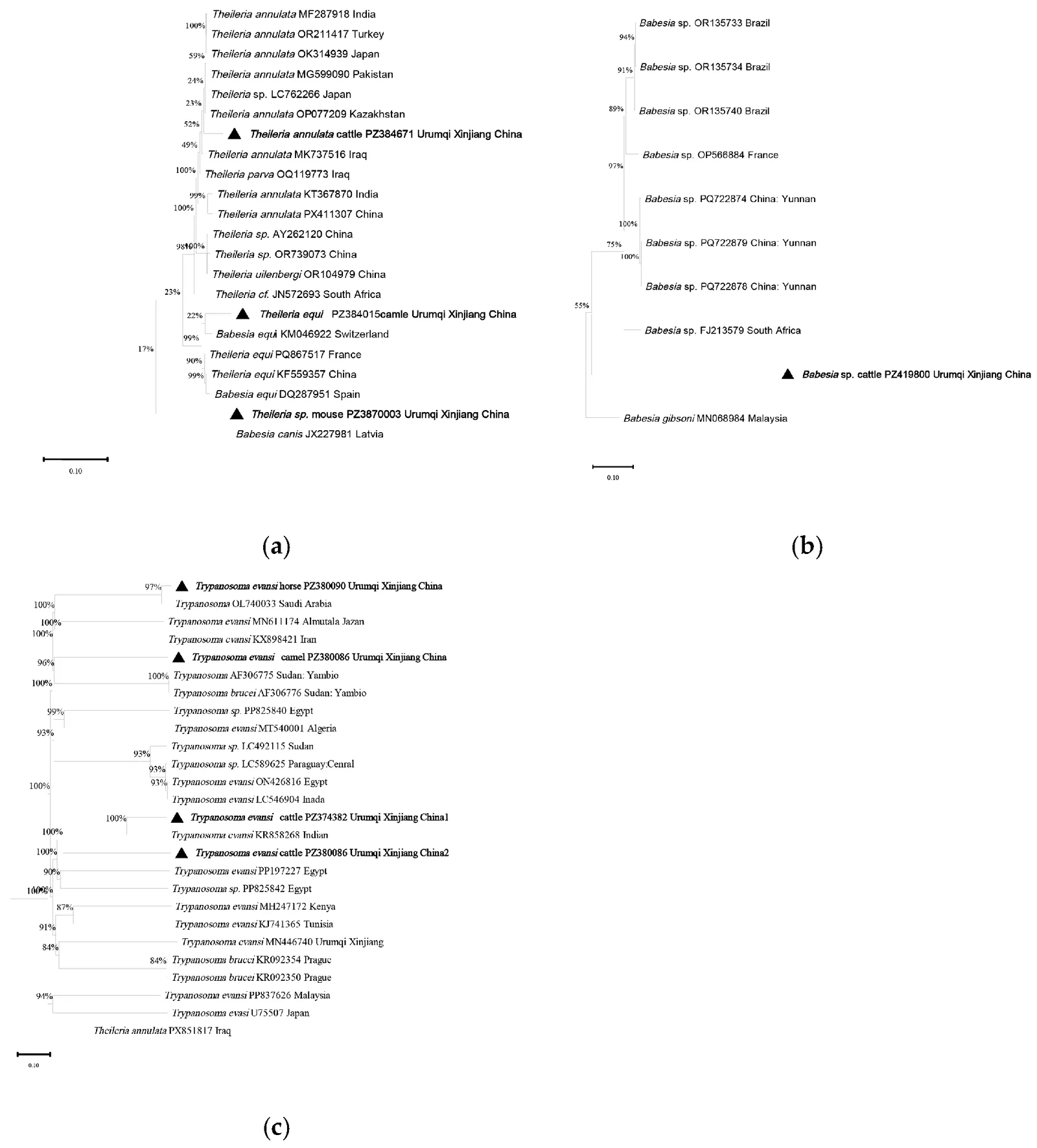
Phylogenetic analysis of different pathogens in various blood samples. (**a**). Phylogenetic relationships of *Theileria* sp. (**b**). Phylogenetic relationships of *Babesia* spp. (**c**). Phylogenetic relationships of *Trypanosoma* spp.

Sequencing of the purified PCR products confirmed that the Theileria species circulating in camels, cattle, and wild mice were predominantly *T. annulata* and *T.equi.* Specifically, the bovine *T. annulata* isolate (PZ384671) shared high genetic identity with an isolate from Kazakhstan (OP077209), while the camel Theileria isolate (PZ384015) was closely related to a *T*. *equi* isolate from Switzerland (KM046922) (Figure 4a). The Babesia isolates obtained in this study formed a single, distinct phylogenetic clade (Figure 4b). All Trypanosoma isolates were identified as *T.evansi.* Phylogenetic analysis indicated that the camel *T*. *evansi* isolate (PZ374382) and the horse *T. evansi* isolate (PZ380090) were closely related to reference strains from Saudi Arabia (AF06775, OL740033), sharing 96–97% sequence identity (Figure 4c). The bovine *T. evansi* isolate (PZ380086) exhibited 100% sequence identity with an Indian isolate (KR858268) and clustered with it in the same phylogenetic tree (Figure 4c).

## 4. Discussion

Xinjiang, located in northwestern China, is characterized by a complex climate and diverse landscapes that create ideal ecological niches for the survival and reproduction of hematophagous arthropods [25]. Tabanids, stable flies, and ixodid ticks, the principal vectors of *Trypanosoma*, *Babesia*, and *Theileria*, proliferate extensively during the grazing season. Their seasonal abundance and prolonged activity during this period likely facilitate the transmission of pathogens among co-grazing livestock and wildlife [26,27,28,29]. Against this ecological backdrop, our results provide the first comprehensive molecular evidence for the circulation of Trypanosoma, Babesia, and Theileria across multiple host species in Xinjiang. The overall PCR-based prevalence of these hemoprotozoan parasites was 46.77% (145/310) among the sampled animals. This high prevalence indicates that these infections are widespread and emphasizes the need for strengthened surveillance across this important pastoral region.

The high prevalence observed in this study also highlights the importance of using sensitive diagnostic methods for the detection of hemoprotozoan infections. A marked discrepancy was observed between molecular and microscopic detection. PCR identified 46.77% (145/310) of samples as positive, whereas Giemsa-stained microscopy detected parasites in only 1.29% (4/310), representing a more than 36-fold difference in detection sensitivity. A similar pattern has been reported in a study of camel trypanosomiasis in Pakistan, in which PCR (14.8%) also considerably outperformed microscopy (8.3%) [30]. This difference is primarily attributable to low parasite burdens in subclinically and chronically infected animals. Technical limitations and difficulties in distinguishing morphologically similar species may have further contributed to the difference. During chronic infections, peripheral parasitemia often falls below the microscopic detection threshold, which is generally considered to be around 10^5^ parasites/mL. Additionally, parasites may be unevenly distributed across blood smears, further complicating visual detection. In contrast, PCR amplifies target nucleic acids regardless of parasite viability or morphological integrity, enabling detection of single-digit copies per microliter of blood [31,32]. Our findings suggest that relying solely on conventional microscopy may substantially underestimate the prevalence of haemoprotozoan infections in Xinjiang. Therefore, PCR-based molecular detection should be regarded as an indispensable diagnostic approach for epidemiological surveillance, particularly for *T*. *evansi* infections, which frequently present as subclinical cases with low parasitaemia.

Beyond these diagnostic nuances, the three detected pathogens exhibited markedly distinct host-associated distribution patterns, suggesting differences in host susceptibility, management practices, and vector exposure. The prevalence of Trypanosoma infection exhibited marked host specificity: it was highest in camels (86.36%), moderate in cattle (21.85%) and horses (11.27%), and absent in wild mice. This pronounced host-associated pattern strongly suggests that camels act as the primary maintenance host for local trypanosomes, likely due to their extensive free-ranging behavior and prolonged exposure to biting vectors [7,8]. In addition, local husbandry practices may have further contributed to this distribution pattern. For example, Midong District, one of the major camel-rearing areas in Xinjiang, is situated on the arid southern fringe of the Gurbantünggüt Desert, where tabanid flies and other biting insects are abundant during the grazing season [33]. Extensive free-range grazing without routine chemoprophylaxis may increase camel exposure to these vectors, thereby facilitating transmission. Consistent with this observation, Midong District (Urumqi) recorded the highest regional Trypanosoma positivity (86.36%, 38/44; Table 2). In contrast, cattle and horses are more commonly stall-fed and managed under semi-intensive or enclosed production systems and receive routine insecticide or acaricide treatments [34,35], which may reduce their exposure to hematophagous vectors. The detection of Trypanosoma in Shuanghe City (31%, 31/100) and Ili (5.6%, 1/18), further indicates its presence across multiple pastoral regions of Xinjiang. The proximity of these areas to Pakistan suggests that transboundary animal movements through regional trade routes may contribute to local positivity rates. Therefore, continued molecular surveillance and cross-border collaboration are warranted to monitor the spread and genetic diversity of *T*. *evansi*.

The striking contrast in Theileria prevalence—highest in wild mice (75%) but completely absent in horses—suggests strong host specificity, likely driven by differences in exposure to infected tick vectors or intrinsic susceptibility (Table 3). The high prevalence observed in wild mice also suggests that rodents may contribute to the local maintenance of Theileria transmission cycles. Rodents are recognized as important hosts for larval and nymphal stages of *Dermacentor*, *Haemaphysalis*, and *Rhipicephalus* ticks [36,37]. Although rodents generally have relatively small home ranges and limited long-distance dispersal, their high population density and capacity to maintain persistent infections may facilitate the circulation of Theileria within local tick populations. The Theileria prevalence in cattle (9.27%) was dominated by *T*. *annulata*, which is consistent with the region-wide occurrence of its principal vector, *Hyalomma* ticks, across Xinjiang’s pastoral ecosystems [16]. In contrast, Babesia infections were detected only in cattle (9.27%) and horses (2.82%), with no infections detected in camels and wild mice. This distribution may reflect differences in host susceptibility, vector ecology, and the host preferences of Babesia-competent tick species. Collectively, these distinct host-associated distribution patterns suggest that surveillance and control strategies should be tailored to the predominant pathogen-host combinations within different livestock production systems. Mixed infections, which pose a substantially greater clinical challenge than single infections, were highly prevalent in this study. Mixed infections occurred in 8.39% (26/310) of sampled animals, with Trypanosoma + Theileria (4.52%) and Trypanosoma + Babesia (3.87%) as the predominant combinations. This highlights the frequent co-exposure of livestock to multiple vector groups in Xinjiang’s pastoral systems (Table 3). Cattle were the most affected species, which likely reflects their simultaneous exposure to tick vectors (carrying Theileria and Babesia) and haematophagous flies (transmitting Trypanosoma) under free-grazing and mixed-farming conditions. This ecological overlap between different vector habitats, combined with management practices, may facilitate concurrent transmission. The triple-infection rate was extremely low (0.32%). This suggests that although multi-pathogen exposure is common, co-infection with three haemoprotozoans may be constrained by vector competence, host immunity, or interspecific pathogen competition. The frequency of co-infections observed here is comparable to the 17.7% reported in livestock from India using multiplex PCR [38]. The clinical impact of these co-infections is highly consequential, as hemoprotozoan co-infections have been associated with enhanced disease severity and more complex clinical manifestations. Experimental and field studies suggest that interactions among hemoprotozoan parasites may alter host immune responses, including suppression of T-cell proliferation and reduced MHC-II expression [39,40], thereby increasing susceptibility to opportunistic pathogens. Furthermore, simultaneous infection with multiple erythrocyte-associated parasites may intensify haemolysis and contribute to anaemia and organ dysfunction [41], potentially resulting in poorer clinical outcomes. Consequently, regional diagnostic workflows should not focus solely on single-pathogen detection. Instead, multiplex PCR or other molecular diagnostic approaches may improve the detection of co-infections, particularly in cattle presenting with non-specific febrile illnesses or chronic infections with low parasite burdens.

Complementing these distribution patterns, phylogenetic analysis confirmed species identities and provided insights into their genetic relationships with isolates reported from other geographic regions, highlighting potential introduction routes into Xinjiang. All trypanosome isolates were identified as *T.evansi*; however, they displayed notable intra-species genetic heterogeneity depending on the host of origin. Specifically, the bovine isolate (PZ380086) showed 100% sequence identity with an Indian strain (KR858268), whereas the camel (PZ374382) and equine (PZ380090) isolates shared >96% sequence similarity with strains reported from Saudi Arabia (AF06775, OL740033) (Figure 4c). These phylogenetic relationships suggest that distinct *T. evansi* lineage has entered Xinjiang via at least two independent routes: a South Asian lineage linked to cattle trade, and a Middle Eastern/Central Asian transmission corridor associated with camel and horse movements [42]. Among the piroplasms, the bovine *T. annulata* isolate (PZ384671) clustered closely with a Kazakhstani strain (OP077209). This close relationship points to a shared ecological distribution of *Hyalomma* ticks across the arid regions of Central Asia [43]. Similarly, the camel *T. equi* isolate (PZ384015) showed close genetic affinity to a Swiss strain (KM046922). This suggests that international movement, particularly through the horse trade, has facilitated parasite dissemination [44]. Collectively, these phylogenetic findings indicate that the hemoprotozoan populations circulating in Xinjiang are genetically diverse and share evolutionary relationships with isolates from multiple regions. This emphasizes the need for continued molecular surveillance, along with effective quarantine and biosecurity measures, to limit the introduction and spread of hemoprotozoan parasites.

Limitations of this study should be acknowledged to guide future research. When interpreting the findings of this study, the potential limitations of the sample composition should be considered. Although the total sample size reached 310 animals, the study involved several different species, resulting in relatively small subgroup sizes for each species. Such uneven distribution of samples across species may, to some extent, affect the statistical power of subgroup analyses and limit the generalisability of our conclusions to specific species. Therefore, future research should expand the sample size for individual or selected species to further validate the present findings. Although 16 samples (5.16%) tested positive for Babesia, species-level identification was not achieved due to the limited resolution of the 18S rRNA marker used in this study. Future studies should incorporate species-specific primers (e.g., for *B. caballi* or *B. bigemina*) to clarify the circulating species [45]. Additionally, entomological investigations were not conducted alongside animal sampling. As a result, information on the abundance, species composition, and infection status of tabanid flies and ticks in the study areas was unavailable. Consequently, direct assessment of vector involvement and transmission intensity was not possible. The cross-sectional design of this study provided only a snapshot of infection status and did not allow assessment of seasonal or temporal variation in pathogen transmission. Longitudinal studies incorporating repeated sampling across grazing and non-grazing seasons would provide a better understanding of infection dynamics. Future research should also integrate vector surveillance, quantitative real-time PCR to estimate parasite burden, and seroconversion follow-up in longitudinal cohorts to comprehensively understand transmission dynamics. Such efforts will provide stronger scientific evidence for the development of early warning systems and targeted intervention strategies against blood protozoan infections in Xinjiang.

## 5. Conclusions

This study demonstrates that Xinjiang serves as a critical ecological intersection for haemoprotozoan transmission among domestic livestock and synanthropic rodents. The distinct host-specific infection patterns observed—particularly the marked differences in susceptibility between camels, cattle, horses, and wild mice underscore the existence of diverse, compartmentalized transmission cycles that are heavily shaped by local agroecological niches. Frequent occurrence of mixed-species infections complicates clinical diagnosis, and supports the routine use of multiplex molecular assays rather than conventional microscopy. Phylogenetic analysis revealed close genetic relationships between local isolates and strains reported from South, Central, and West Asia indicating connections between haemoprotozoan populations in Xinjiang and those in other regions. These findings highlight the need for an integrated, transboundary surveillance framework that combines wildlife monitoring with livestock health management to effectively mitigate epizootic risks across Eurasia.

## Figures and Tables

**Table 1:** List of primers for molecular identification and pathogen detection.

Pathogen	Primers (5′-3′)	PCR Conditions	Fragment (bp)	Genetic Markers and References
*Trypanosoma* spp.	CCGGAAGTTCACCGATATTG TTGCTGCGTTCTTCAACGAA	Initial denaturation: 95 °C for 5 min followed by 35 cycles of denaturation at 95 °C for 30 s, annealing at 54.2 °C for 30 s, extension at 72 °C for 48 s, and final extension at 72 °C for 5 min.	250–700	ITS-1 [19]
*Babesia* spp.	CATTACAACAGTTATAGTTTCTTTGG CTAGGCATTCCTCGTTCATGATTTAG	Initial denaturation: 95 °C for 5 min followed by 35 cycles of denaturation at 95 °C for 30 s, annealing at 52.2 °C for 30 s, extension at 72 °C for 84 s, and final extension at 72 °C for 5 min.	1400	18S rRNA [20]
*T. annulata* (Cattle)	TTCGTTATCTCATCCGTT ATACTTGGCATTGTTTGG	Initial denaturation: 95 °C for 5 min followed by 35 cycles of denaturation at 95 °C for 30 s, annealing at 46.8 °C for 30 s, extension at 72 °C for 32.1 s, and final extension at 72 °C for 5 min.	534	Tams-1 [21]
*Theileria* (Camel/Mice)	AGACAGAGGAAGGATTGACA CTGATGACTTGCGCATACTA	Initial denaturation: 95 °C for 5 min followed by 35 cycles of denaturation at 95 °C for 30 s, annealing at 52.1 °C for 30 s, extension at 72 °C for 23.2 s, and final extension at 72 °C for 5 min.	386	18S rRNA [22]
*Theileria* (Horse)	CGAAGACGATCAGATACCGTCG TGCCTTAAACTTCCTTGCGAT	Initial denaturation: 95 °C for 5 min followed by 35 cycles of denaturation at 95 °C for 30 s, annealing at 54 °C for 30 s, extension at 72 °C for 26 s, and final extension at 72 °C for 5 min.	430	18S rRNA [23]

**Table 2:** Detection rates of haemoprotozoan pathogens in blood samples from different regions.

Area	No. of Blood Samples	No. of Infection (%; 95% CI)		
		*Trypanosoma* spp.	*Babesia* spp.	*Theileria* spp.
Urumqi	44	38 (86.36; 72.9–93.8)	-	3 (6.82; 2.3–18.2)
Changji	44	-	-	33 (75; 60.6–85.4)
Turpan	51	2 (3.92; 1.1–13.2)	13 (25.5; 15.6–38.9)	13 (25.5; 15.6–38.9)
lli	18	1 (5.6; 1.0–25.8)	2 (11.1; 3.1–32.8)	-
Shuanghe	100	31 (31; 22.8–40.6)	1 (1; 0.2–5.5)	1 (1; 0.2–5.5)
Bayingolin	53	7 (13.2; 6.6–24.9)	-	-
total	310	79 (25.48; 21.0–30.6)	16 (5.16; 3.2–8.2)	50 (16.13; 12.5–20.6)

“-” means not detected. The table uses Graphpad Prism 10.0 software.

**Table 3:** Detection rates of pathogens in blood samples from different animals.

Type of Infection	Species	No. of Infection (%; 95% CI)				
		Cattle (*N* = 151)	Horse (*N* = 71)	Camel (*N* = 44)	Wild Mouse (*N* = 44)	Total (310)
Single infection	*Trypanosoma* spp.	33 (21.85; 16.01–29.10)	8 (11.27; 5.82–20.69)	38 (86.36; 73.29–93.60)	-	79 (25.48; 20.95–30.61)
	*Theileria* spp.	14 (9.27; 5.60–14.96)	-	3 (6.82; 2.35–18.23)	33 (75; 60.56–85.43)	50 (16.13; 12.45–20.63)
	*Babesia* spp.	14 (9.27; 5.60–14.96)	2 (2.82; 0.78–9.70)	-	-	16 (5.16; 3.20–8.22)
Dual infection	*Trypanosoma* spp.+ *Theileria* spp.	12 (7.95; 4.60–13.38)	-	2 (4.55; 1.26–15.13)	-	14 (4.52; 2.71–7.44)
	*Trypanosoma* spp.+ *Babesia* spp.	12 (7.95; 4.60–13.38)	-	-	-	12 (3.87; 2.23–6.64)
Triple infection	*Trypanosoma* spp. + *Theileria* spp. + *Babesia* spp.	1 (0.66; 0–1.96)	-	-	-	1 (0.32; 0–0.95)

“-” means not detected. The table uses Graphpad Prism 10.0 software.

## Data Availability

The original contributions presented in this study are included in the article. Further inquiries can be directed to the corresponding authorsSee the appendix for some PCR test result images of *Babesia* spp., *Theileria* spp., and *Trypanosoma* spp.

## Abbreviations

The following abbreviations are used in this manuscript:

*T*. *evansi*	*Trypanosoma evansi*
*T*. *annulata*	*Theileria annulata*
*T*. *equi*	*Theileria equi*
